# A Study on L-Asparaginase of *Nocardia levis* MK-VL_113

**DOI:** 10.1100/2012/160434

**Published:** 2012-04-24

**Authors:** Alapati Kavitha, Muvva Vijayalakshmi

**Affiliations:** Department of Botany and Microbiology, Acharya Nagarjuna University, Guntur 522 510, India

## Abstract

An enzyme-based drug, L-asparaginase, was produced by *Nocardia levis* MK-VL_113 isolated from laterite soils of Guntur region. Cultural parameters affecting the production of L-asparaginase by the strain were optimized. Maximal yields of L-asparaginase were recorded from 3-day-old culture grown in modified asparagine-glycerol salts broth with initial pH 7.0 at temperature 30°C. Glycerol (2%) and yeast extract (1.5%) served as good carbon and nitrogen sources for L-asparaginase production, respectively. Cell-disrupting agents like EDTA slightly enhanced the productivity of L-asparaginase. Ours is the first paper on the production of L-asparaginase by *N. levis*.

## 1. Introduction

L-asparaginase (L-asparagine amino hydrolase EC 9.5.1.1) is a potent antitumor enzyme that catalyzes the hydrolysis of L-asparagine to L-aspartic acid and ammonium ion. This enzyme has been widely exploited in the treatment of certain kinds of cancer especially acute lymphoblastic leukaemia since the time it was obtained from *Escherichia coli *and its antineoplastic activity demonstrated in guinea pig serum [1–3]. Due to its prompt therapeutic potential, screening of microbial sources for asparaginase activity has been greatly intensified and well documented in *E*.* coli *[4–6], *Erwinia carotovora *[7, 8], *Enterobacter aerogenes *[9], *Corynebacterium glutamicum *[10], *Candida utilis *[11], *Serratia marcescens *[12], *Staphylococcus aureus *[13], and *Thermus thermophilus *[14]. In addition, actinomycetes act as potential candidates for the production of L-asparaginase. Particularly, *Mycobacterium tuberculosis *[15], *Streptomyces griseus *[16], *S*. *karnatakensis*, *S*. *venezuelae *[17], *S*. *longsporusflavus *F-15 [18], *S*. *phaeochromogenes *FS-39 [19], and* Nocardia asteroides* [20] were proved to be potential producers of this enzyme. However, very little information is available on the production of L-asparaginase by the genus *Nocardia*. While screening the actinomycetes for asparaginase production, a strain with good asparaginase activity on asparagine- glycerol salts agar medium was identified as *Nocardia levis *by 16S rRNA analysis. In the present study, an attempt has been undertaken to reveal the optimization of L-asparaginase production by *N*. *levis*.

## 2. Material and Methods

*Nocardia levis *MK-VL_113 was isolated from laterite soil samples of Guntur region, and the 16S rRN A gene sequence of the strain has been deposited in NCBI genbank with an accession number FJ209734 [21].

### 2.1. Production Profile of L-Asparaginase

For determining the production profile of L-asparaginase, culture suspension prepared from one-week-old culture of the strain was inoculated into asparagine-glycerol salts (ISP-5) broth containing 1% asparagine, 1% glycerol, 0.1% K_2_HPO_4_, 0.1% trace salt solution with initial pH 7.2. The inoculated flasks were incubated at 35°C for 6 days in order to estimate the cell growth of the strain as well as L-asparaginase production for every 24 h interval. Cell growth was expressed in terms of dry weight of biomass (mg/mL).

L-asparaginase assay was performed according to the procedure described by Peterson and Ciegler [22] with slight modifications. Cells were harvested by centrifuging the culture broth at 10,000 rpm for 15 min and were ground in Tris-HCl buffer by using homogenizer. Later, it was again centrifuged, and the cell free extract (0.2 mL) obtained was mixed with 0.8 mL of 0.05 M Tris-HCl buffer and 1 mL of 0.04 M L-asparagine. After incubating the reaction mixture for 15 min at 37°C in a water bath shaker, the reaction was terminated by the addition of 0.5 mL of 15% (w/v) trichloroacetic acid. Precipitated proteins were removed by centrifugation, and the liberated ammonia was determined spectrometrically at 500 nm by nesslerization. Tubes kept at zero time incubation served as control. Enzyme activity was determined on the basis of liberation of ammonia calculated with reference to a standard curve of ammonium sulphate. One L-asparaginase unit (IU) equals to that amount of enzyme which releases 1 *μ*M of ammonia (ammonium sulphate as standard) in 1 min at 37°C. The cell dry weight was recorded simultaneously by drying the cell debris collected after centrifugation in an oven at 90°C for 24 h.

### 2.2. Optimization of L-Asparaginase Production

Influence of different cultural conditions such as initial pH, temperature, carbon, and nitrogen sources on the production of L-asparaginase was determined.

#### 2.2.1. Initial pH and Temperature

Impact of pH on the production of L-asparaginase was examined by culturing the strain in ISP-5 broth adjusted to various pH levels ranging from 4.0 to 10.0. The optimal pH achieved at this step was used for further study. To determine the optimum temperature for L-asparaginase production, the strain was cultured in ISP-5 broth at different temperatures, namely, 20° to 40°C for 72 h of incubation.

#### 2.2.2. Carbon Sources

To investigate the effect of carbon sources on L-asparaginase production by the strain, ISP-5 broth was supplemented with different carbon sources such as arabinose, fructose, galactose, glucose, lactose, maltose, mannitol, sorbitol, starch, sucrose, and xylose each at a concentration of 1% (w/v). Impact of different concentrations of best carbon source (1–4% w/v) on L-asparaginase activity of the strain was studied.

#### 2.2.3. Nitrogen Sources

Different nitrogen sources, namely, L-asparagine, beef extract, L-glutamine, malt extract, peptone, tryptone, and yeast extract were added at a rate of 1% (w/v) to ISP-5 broth consisting an optimal amount of superior carbon source. Besides, the optimal concentration of nitrogen source (1–3% w/v) supporting high yields of L-asparaginase production was determined by maintaining all other conditions at optimum levels [23].

#### 2.2.4. Effect of Cell-Disrupting Agents

The effect of cell disrupting agents like EDTA, lysozyme, penicillin-G, and SDS on the release of L-asparaginase from the 72 h old cultures was tested [24]. Harvested cells suspended in 0.05 M Tris-HCl buffer (pH 7.5) were centrifuged at 10,000 rpm for 15 min. The resulting pellet was washed twice and resuspended in the same buffer. Cell-disrupting agents (50 *μ*g/mL) prepared separately in Tris-HCl were used to treat the cells twice and centrifuged. The cell debris was discarded, and the supernatant (0.2 mL) thus collected was used as enzyme source.

## 3. Results and Discussion

### 3.1. Production Profile of L-Asparaginase

**Figure 1:**
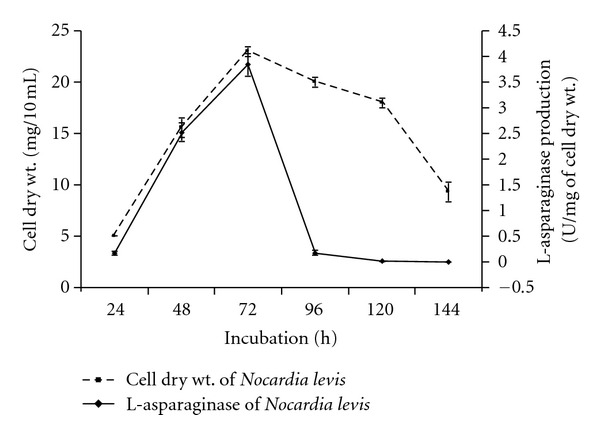
Production profile of L-asparaginase by *Nocardia levis *MK-VL_113 grown in modified ISP-5 broth. (Values are the means of three replicates ±  SD.)

Production of L-asparaginase by *N*. *levis *started after 24 h of cell growth. Maximum levels of L-asparaginase production as well as cell growth were observed after 72 h of incubation (Figure 1). It revealed a good correlation between enzymatic yields and active cell growth of the strain. A positive correlation between the cell growth and L-asparaginase productivity was reported in *S*. *karnatakensis *[25] and *S*. *albidoflavus *[23]. In *Amycolatopsis *CMU-H002, maximum yields of L-asparaginase and biomass were obtained from 72 h old culture [26].

### 3.2. Optimization of L-Asparaginase Production

#### 3.2.1. Initial pH and Temperature

**Figure 2:**
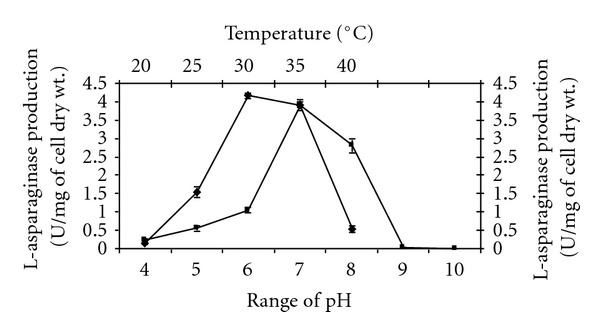
Effect of initial pH and temperature on L-asparaginase production by *Nocardia levis *MK-VL_113 cultured in modified ISP-5 broth. (Values are the means of three replicates ±  SD.)

A study of initial pH levels (4–10) on the production of L-asparaginase by the strain indicated optimal enzyme activity at pH 7.0 (Figure 2). These findings are in conformity with the results of Koshy et al. [27], Abdel-All et al. [28] and Dhevendaran and Anithakumar [29] who recorded pH 7.0 as optimum for L-asparaginase production in *S*. *longsporusflavus *F-15, *S*. *plicatus*, *S*. *phaeochromogenes *FS-39, and *Streptomyces *AQB VC67, respectively. Other actinomycetes species such as *S*. *aureofasciculus *LA-2, *S*. *chattanoogenesis *LA-8, *S*. *hawaiiensis *LA-15, *S*. *orientalis *LA-20, *S*. *canus *LA-29 and *S*. *olivoviridis *LA-35 exhibited optimum growth and L-asparaginase production at pH 7 to 8 [30]. 

Impact of temperature on the production of L-asparaginase by the strains is presented in Figure 2. Production of L-asparaginase was high with the strain cultured in modified ISP-5 broth at 30°C. In *S*. *collinus *[17] and *S*. *longsporusflavus *F-15 [18], the optimum temperature recorded was 30°C for L-asparaginase production. Koshy et al. [27] reported 29 ± 2°C as optimum temperature for L-asparaginase production in *S*. *plicatus*. The present study revealed that L-asparaginase production by the strain was high when grown in modified ISP-5 broth with initial pH 7.0 for 72 h at 30°C.

#### 3.2.2. Carbon Sources

Different carbon sources like arabinose, fructose, galactose, glucose, lactose, maltose, mannitol, sorbitol, starch, sucrose, and xylose were amended in ISP-5 broth to determine their impact on L-asparaginase production. As compared to other carbon sources tested, L-asparaginase production was high in the basal medium containing glycerol (Table 1). Krishna Reddy and Reddy [31] also noted glycerol as one of the best carbon sources for L-asparaginase activity of *S*. *albus*.

The final pH of the fermentation broth consisting glycerol became alkaline whereas in media amended with other carbon sources became acidic that may lead to decline in productivity of L-asparaginase. Acidity of fermentation medium could inhibit the production of L-asparaginase [32], and, because of this nature, glucose is reported as a repressor for L-asparaginase synthesis [9, 23, 33].

Besides, the effect of different concentrations of glycerol (1–4%) on L-asparaginase production is recorded (Table 2). Optimal yields of L-asparaginase by the strain were achieved in the medium amended with 2% glycerol, whereas its biosynthesis greatly declined with further hike in carbon source. The optimal level of carbon source for L-asparaginase production by *S*. *phaeochromogenes *FS-39 was 2% glycerol [28], while it was 1.5% starch for *S*. *longsporusflavus *F-15 [34]. In the present work, optimal yields of L-asparaginase were recorded by culturing *N*. *levis *in the modified ISP-5 broth containing 2% glycerol with initial pH 7.0 at 30°C for 72 h.

#### 3.2.3. Nitrogen Sources

The effect of nitrogen compounds on the production of L-asparaginase by the strain was studied by incorporating different nitrogen sources to modified ISP-5 broth containing 2% glycerol. L-asparaginase production by the strain varied with different nitrogen compounds tested (Table 3). Among them, culture medium amended with yeast extract favored maximal L-asparaginase production by the strain followed by tryptone. In *Erwinia aroideae*, yeast extract supported high yields of L-asparaginase production [35] while tryptone and yeast extract stimulated the production of this enzyme in *E*. *carotovora *EC-113 [7]. L-asparagine was reported to be suitable nitrogen source for L-asparaginase production by *S*. *collinus *[17] and *S*. *karnatakensis *and *S*. *venezuelae *[25]. Optimization studies of Abdel-All et al. [28] indicated glycerol L-asparaginase yeast extract (GAY) as a fruitful medium for the synthesis of L-asparaginase by *S*. *phaeochromogenes *FS-39.

The optimal level of yeast extract was found to be 1.5% for obtaining high amounts of L-asparaginase (Table 4). A gradual decline in L-asparaginase production was found with further rise in yeast extract levels. Yeast extract is essential for cell growth and L-asparaginase synthesis, but, in high concentrations, it inhibits the production of L-asparaginase [36]. In *S*. *albidoflavus*, Narayana et al. [23] recorded 2% yeast extract as optimal concentration for L-asparaginase production. In the present study, enhanced levels of L-asparaginase production by *N*. *levis *was recorded in the modified ISP-5 broth containing 1.5% yeast extract and 2% glycerol with initial pH 7.0 incubated at 30°C for 72 h.

#### 3.2.4. Effect of Cell-Disrupting Agents on L-Asparaginase Production

Production of L-asparaginase by the strain was examined for its intracellular or extracellular nature. Enzyme production was observed only in cell extracts. Therefore, the effect of cell-disrupting agents like EDTA, lysozyme, penicillin-G, and SDS on the release of L-asparaginase from the cells was determined. Among the cell disrupting agents tested, EDTA was found to be more effective for the release of L-asparaginase from the cells (Table 5). In *S*. *griseus *[16] and *S*. *karnatakensis *[37], the production of L-asparaginase was found to be intracellular. Enhanced levels of L-asparaginase were detected in *S*. *albidoflavus* when treated with cell disrupting agents like EDTA [23].

In the present study, the optimal cultural and nutritional conditions for the production of L-asparaginase by *N*. *levis *MK-VL_113 were recorded. Biosynthesis of L-asparaginase by the strain was high when cultured in modified ISP-5 broth composing glycerol (2%) and yeast extract (1.5%) with initial pH 7.0 at temperature 30°C. EDTA was proved to be an efficient cell-disrupting agent in the release of intracellular enzyme from the cells of the strain. This is the new report on the production of L-asparaginase by *N*. *levis*.

## Figures and Tables

**Table 1:** L-asparaginase production by *Nocardia levis *grown in modified ISP-5 broth amended with different carbon sources.

Carbon sources (1% w/v)	L-asparaginase production (U/mg of cell dry wt.)
*Control	4.15 ± 0.063
Arabinose	4.03 ± 0.16
Fructose	3.9 ± 0.223
Galactose	3.75 ± 0.221
Glucose	0.32 ± 0.016
Lactose	1.05 ± 0.064
Maltose	0.4 ± 0.057
Mannitol	0.36 ± 0.013
Sorbitol	0.09 ± 0.004
Starch	1.32 ± 0.131
Sucrose	0.19 ± 0.013
Xylose	0.57 ± 0.042

Values are the means of three replicates ±  SD. *Modified ISP-5 broth with 1% glycerol (v/v).

**Table 2:** Carbon levels on L-asparaginase production by *Nocardia levis *in modified ISP-5 broth.

Glycerol (v/v) concentrations	L-asparaginase production (U/mg of cell dry wt.)
1%	4.15 ± 0.063
2%	4.28 ± 0.016
3%	1.23 ± 0.009
4%	0.06 ± 0.004

Values are the means of three replicates ±  SD.

**Table 3:** L-asparaginase production by *Nocardia levis *grown in modified ISP-5 broth amended with different nitrogen sources.

Nitrogen sources (1% w/v)	L-asparaginase production (U/mg of cell dry wt.)
L-asparagine	4.28 ± 0.016
Beef extract	0.64 ± 0.061
L-glutamine	4.19 ± 0.008
Malt extract	2.47 ± 0.075
Peptone	3.86 ± 0.154
Tryptone	4.34 ± 0.076
Yeast extract	4.56 ± 0.172

Values are the means of three replicates ±  SD.

**Table 4:** Impact of yeast extract concentrations on L-asparaginase production by *Nocardia levis *grown in modified ISP-5 broth.

Yeast extract concentrations (w/v)	L-asparaginase production (U/mg of cell dry wt.)
1%	4.56 ± 0.172
1.5%	5.06 ± 0.002
2%	3.42 ± 0.142
2.5%	1.62 ± 0.043
3%	0.97 ± 0.013

Values are the means of three replicates ±  SD.

**Table 5:** Effect of cell-disrupting agents on the release of L-asparaginase from 72 h old culture of *Nocardia levis. *

Cell disrupting agents (50 *μ*g/ml)	Release of L-asparaginase from the culture (U/mg of cell dry wt.)
*Control	5.06 ± 0.002
EDTA	5.34 ± 0.04
Lysozyme	5.29 ± 0.019
Penicillin-G	5.11 ± 0.082
SDS	4.01 ± 0.163

Values are the means of three replicates ±  SD. *Cell disruption using homogenizer.
